# Variation in COVID-19 Diagnosis by Zip Code and Race and Ethnicity in Indiana

**DOI:** 10.3389/fpubh.2020.593861

**Published:** 2020-12-11

**Authors:** Amy E. Hanson, David S. Hains, Andrew L. Schwaderer, Michelle C. Starr

**Affiliations:** Department of Pediatrics, Indiana University, Indianapolis, IN, United States

**Keywords:** COVID-19, disparities (health, racial), social determinants of health, SARS-CoV 2, coronavirus

## Abstract

**Objectives:** To describe variations in coronavirus disease 2019 (COVID-19) diagnosis by zip code race and ethnicity in Indiana.

**Methods:** Cross-sectional evaluation of subjects with SARS-CoV-2 at Indiana University Health. We performed two separate analyses, first evaluating likelihood of COVID-19 diagnosis by race (Caucasian, African American, Asian, or other) and ethnicity (Hispanic vs. non-Hispanic) in the cohort encompassing the entire state of Indiana. Subsequently, patient data was geolocated with zip codes in Marion County and the immediate surrounding counties, and descriptive statistical analyses were used to calculate the number of COVID-19 cases per 10,000 persons for each of these zip codes.

**Results:** Indiana had a total of 3,892 positive COVID-19 cases from January 1 to April 30, 2020. The odds of testing positive for COVID-19 were four-fold higher in African Americans than non-African Americans (OR 4.58, 95% CI 4.25–4.94, *P* < 0.0001). Increased COVID-19 cases per 10,000 persons were seen in zip codes with higher percentage of African American (median infection rate of 17.4 per 10,000 population in zip codes above median % African American compared to 6.7 per 10,000 population in zip codes below median % African American, with an overall median infection rate 9.9 per 10,000 population, *P* < 0.0001) or Hispanic residents (median infection rate of 15.9 per 10,000 population in zip codes above median % Hispanic compared to 7.0 per 10,000 population in zip codes below median % Hispanic, overall median infection rate 9.6 per 10,000 population, *P* < 0.0001).

**Conclusions:** Individuals from zip codes with higher percentages of African American, Hispanic, foreign-born, and/or residents living in poverty are disproportionately affected by COVID-19. Urgent work is needed to understand and address the disproportionate burden of COVID-19 in minority communities and when economic disparities are present.

## Introduction

The novel severe acute respiratory syndrome coronavirus 2 (SARS-CoV-2) has led to a global pandemic. Severe SARS-CoV-2 infection is characterized by rapid and efficient transmission and causes coronavirus disease 2019 (COVID-19) which results in a range of clinical courses including severe acute respiratory distress syndrome, viral pneumonia, mild upper respiratory infection (URIs) and asymptomatic carriers (1, 2). COVID-19 has drawn increased focus on social determinants of health (SDH), which are reflected in geographic locations and are associated with increased COVID-19 disease burden in African American and Hispanic minority populations (3). The most pervasive disparities in COVID-19 diagnosis have been seen in African American and Hispanic individuals, however studies have to date only focused on single studies of dense urban areas (1). Severe COVID-19 presentations occur more frequently in those with chronic diseases and minority populations, most notably African American and/or Hispanic individuals (2). Growing attention is now being focused on disparities in race and ethnicity, as preliminary data suggests that African American individuals and Hispanic individuals may have a disproportionate burden of COVID-19.

Indiana emerged as a United States COVID-19 hotspot in March, 2020, with sustained ongoing transmission and steady rates of infection. Due to its varied demographics (including areas of dense urban, suburban and rural), the state serves as an informative model to study the association between SDH and COVID-19. Growing literature suggests that COVID-19 infection rates by race and ethnicity may not fully be accounted for by disparities in sociodemographic status (4). Differences in access to testing may also manifest in differences in identified cases as well. We sought to better understand the relationship between sociodemographic variables and diagnosis of COVID-19, to better understand the association with race and ethnicity as a first step to address these disparities (4).

We conducted a cross-sectional cohort study in those diagnosed with COVID-19 to assess the rate of diagnosis based on zip code characteristics of race and ethnicity. We hypothesized that African American individuals would have higher rates of COVID-19 than non-African American individuals, and that that Hispanic individuals would have higher rates of COVID-19 than non-Hispanic individuals.

## Methods

We evaluated all patients with positive nasopharyngeal swab for SARS-CoV-2 at all Indiana University Health or Eskenazi Health hospitals from January 1, 2020 to April 30, 2020. Due to the centralized Indiana University health system, this covers a wide range of demographic and geographic populations and is broadly representative of the general population of Indiana. Abstracted electronic medical record (EMR) and billing data was obtained from the Regenstrief Institute following Institutional Review Board approval.

We performed two separate analyses on the larger, and then a smaller sub-cohort. The primary outcome for both analyses was a diagnosis of COVID-19 based on nasopharyngeal swab positive SA RS-CoV-2. We first evaluated the likelihood of COVID-19 diagnosis by patient demographics. We used a primary exposure of race (Caucasian, African-American, Asian, or other) and ethnicity (Hispanic vs. non-Hispanic) and an outcome of COVID-19 diagnosis in the state of Indiana.

We then examined COVID-19 cases based on zip code characteristics in the 108 zip codes of the most populous county, which houses the main hospitals, and eight contiguous counties. The primary exposure was residing zip code, as reported through billing and EMR data. State demographic data available by zip code for counties including and immediately surrounding the IUH and Eskenazi Health main facility locations was used. Zip code data obtained and utilized for comparison in the study included population, percentage African American, percentage Hispanic, percentage foreign-born, and percentage living in poverty as reported by 2019 American Community Survey, an annual nationwide audit conducted by the US Census Bureau.

**Figure 1:**
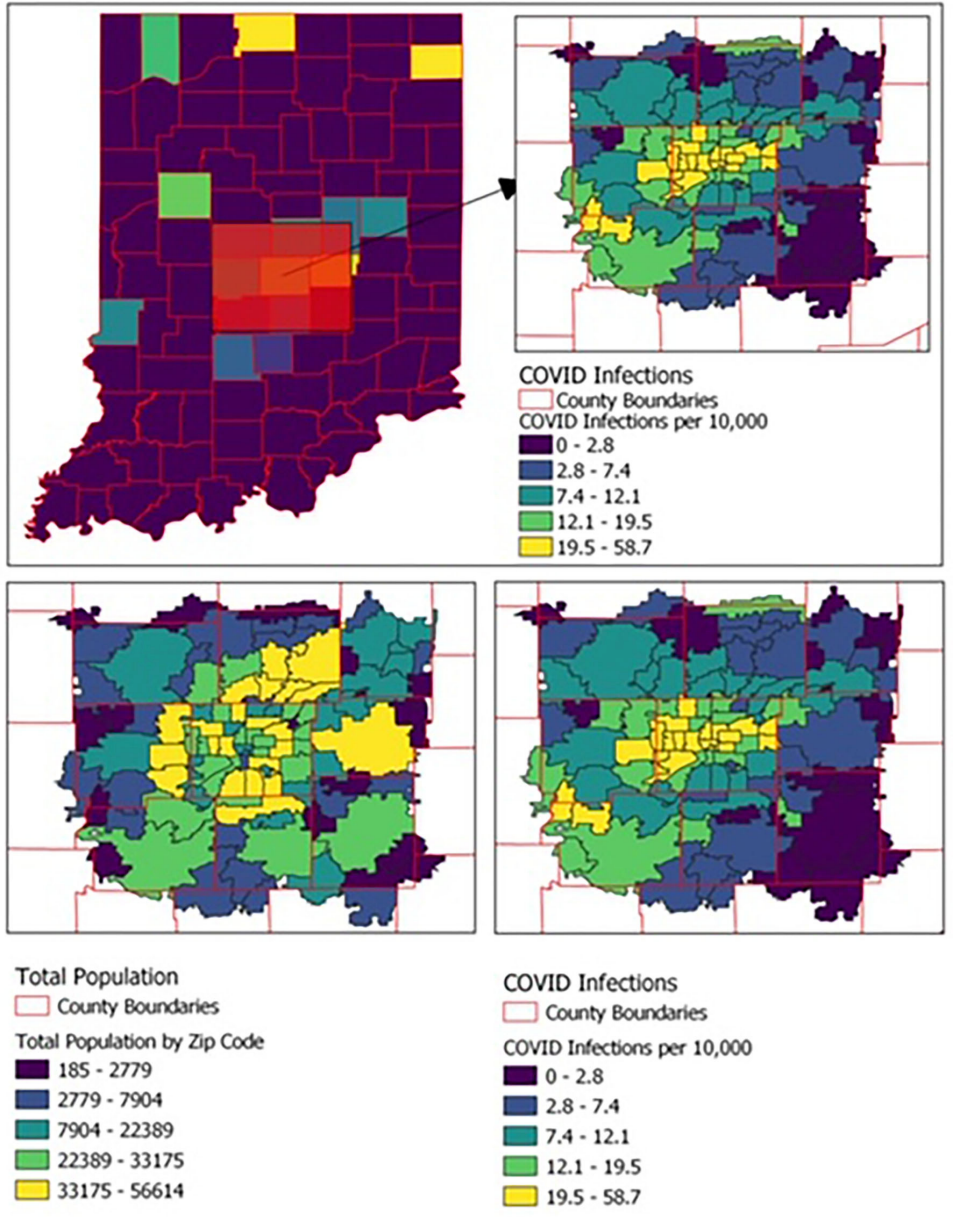
Geographic distribution of COVID-19 cases and population by zip code among the 108 zip codes of the state's most populous county and the eight contiguous counties.

Descriptive statistical analyses were used to calculate the likelihood of COVID-19 based on patient race and ethnicity. Odds ratios were calculated to assess the likelihood of COVID-19 based on race and ethnicity. Patient data was then geolocated with zip codes using the 2019 American Community Survey, an annual nationwide audit conducted by the US Census Bureau (Figure 1). Zip codes were based on an ESRI ArcGIS 10 dataset from Tele Atlas, and maps produced using QGIS 3.10.6.3 (5). We compared COVID-19 diagnosis rate in zip codes with higher percentage of residents (above the median) of each demographic characteristic to those with lower percentage of residents (below the median) with each demographic characteristic. We evaluated Race (Caucasian, African American, Asian, and Other), Ethnicity (Hispanic, non-Hispanic), Foreign-born (Yes, No), Poverty Rate (based on U.S. Census Bureau poverty thresholds), and Geographic Density (based on US Census Bureau zip code data which is calculated by dividing the number of persons in each zip code by the total land area). Categorical variables were compared using chi-square. We calculated Pearson correlation coefficients to assess the correlation between the demographic characteristics and zip code. A two-sided significance level of 0.05 was used for all tests. Statistical analyses were performed using SPSS Statistics, version 25 (IBM, Armonk, New York).

## Results

Indiana had a total of 3,892 positive COVID-19 cases from January 1 to April 30. Of those with COVID-19, 3,081 were from the 108 zip codes of the state's most populous county (which houses the main hospitals) and the eight contiguous counties (Figure 1). Of these 3,081 cases, 182 did not have an associated race and/or ethnicity and were excluded from further analyses.

In the larger statewide cohort, 1,024 (33%) COVID-19-positive individuals were African American and 507 (16%) were Hispanic. This is disparate from the proportion of the Indiana population that these groups constitute, as African Americans and Hispanics make up 8.8% and 4.5% of the Indiana state population, respectively (3). Females were slightly more predominant, with 1,848 (60%) of the positives. The odds of COVID-19 infection was four-fold higher in African Americans than non-African Americans (OR 4.58, 95% CI 4.25–4.94, *P* < 0.0001) and two-fold higher in Hispanics than non-Hispanics (OR 2.58, 95% CI 2.34–2.83, *P* < 0.0001). The median number of COVID-19 infections in the larger cohort, which included all Indiana zip codes, was 0.

**Figure 2:**
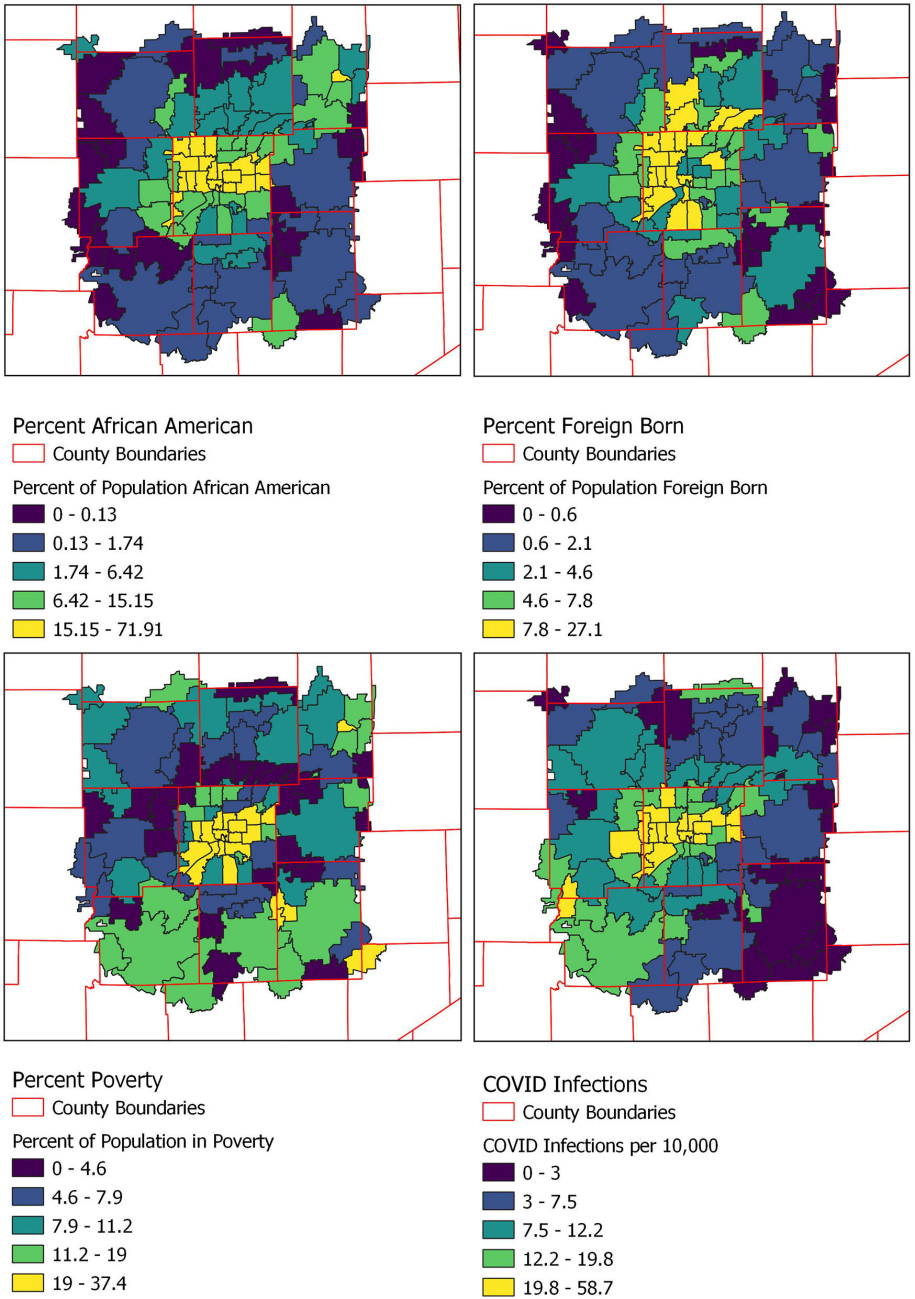
Percentage of population self-identifying as African American, Hispanic, or foreign born and those below the poverty line by zip code.

We then examined COVID-19 cases based on zip code characteristics in the 108 zip codes of the most populous county, which houses the main hospitals and eight contiguous counties. Higher rates of COVID-19 cases per 10,000 persons were seen in zip codes with higher percentage of African American residents (median infection rate of 17.4 per 10,000 population in zip codes above median % black compared to 6.7 per 10,000 population in zip codes below median % black, *P* < 0.0001; Pearson coefficient 0.67, *P* < 0.0001), Hispanic (median infection rate of 15.9 per 10,000 population in zip codes above median % Hispanic compared to 7.0 per 10,000 population in zip codes below median % Hispanic, *P* < 0.0001; Pearson coefficient 0.59, *P* < 0.0001), foreign-born (median infection rate of 13.5 per 10,000 population in zip codes above median % foreign born compared to 6.4 per 10,000 population in zip codes below median % foreign born, *P* < 0.0001; Pearson coefficient 0.52, *P* < 0.0001), or those with more residents living in poverty (median infection rate of 13.5 per 10,000 population in zip codes above median % living in poverty compared to 8.5 per 10,000 population in zip codes below median % living in poverty, *P* < 0.0001; Pearson coefficient 0.49, *P* < 0.0001). Individuals living in more geographically dense zip codes were more likely to have COVID-19 infection (median infection rate 15.0 per 10,000 population in zip codes above median population density compared to 4.5 per 10,000 population in zip codes below median population density, *P* < 0.0001; Pearson coefficient 0.68, *P* < 0.0001) (Table 1, Figure 2).

**Table 1:** Comparison of COVID-19 infection rate per 10,000 persons by sociodemographic characteristics in 108 zip codes^a^ in Indiana, United States.

	**Median infection rate in zip codes above median demographic**	**Median infection rate in zip codes below median demographic**	**Median infection rate of zip codes with available demographic data (Reference)**	***P*-value**	**Pearson correlation coefficient**	***P*-value**
African American residents	17.4	6.7	9.9	<0.0001	0.67	<0.0001
Hispanic residents	15.9	7.0	9.6	<0.0001	0.59	<0.0001
Foreign-born residents	13.5	6.4	9.6	<0.0001	0.52	<0.0001
Geographic density^b^	15.0	4.5	9.4	<0.0001	0.68	<0.0001
Living in poverty	13.5	8.5	9.6	<0.0001	0.49	<0.0001

a *Zip code data obtained and utilized for comparison in the study included population, % black, % Hispanic, % foreign-born, and % living in poverty as reported by 2019 American Community Survey, an annual nationwide audit conducted by the US Census Bureau*.

b *Geographic density (based on US Census Bureau zip code data which is calculated by dividing the number of persons in each zip code by the total land area)*.

## Discussion

In this cross-sectional cohort study of a large and representative sample of subjects with COVID-19 in the Midwest, we found that individuals from zip codes with higher percentages of residents who were African American, Hispanic, foreign-born, living in poverty, and/or living in more geographically dense locations were disproportionately affected by COVID-19. We focused on this smaller cohort of 108 zip codes as this is where our data was concentrated (immediately surrounding our healthcare practice institutions). This choice was further supported by the fact that the median number of COVID-19 cases in all Indiana zip codes was 0, reinforcing our belief that individuals living in zip codes farther away from central Indiana likely presented to local hospitals, if at all, for COVID-19 testing and their data was therefore not available for use in this analysis.

These findings are similar to those reported in the one published single-center study assessing risk factors for COVID-19 diagnosis (6). We note that these increased rates may be in part due to comorbidities associated with higher risk of severe COVID-19-related morbidity including cardiovascular disease, diabetes mellitus and obesity, conditions more prevalent in African American and Hispanic than non-African American and non-Hispanic populations (2, 6–8). However, other factors more related to SDH may have contributed more to these findings. These include disparities in employment which preclude social distancing or working from home, inequalities in transportation which necessitate reliance on public transportation, crowded housing circumstances, inequity of testing access, limited health literacy, or economic disparities which compel return to work despite ongoing sustained community SARS-CoV-2 trasnmission (9).

Our preliminary work finds differences in COVID-19 diagnosis due to multiple factors which we broadly represent by zip code suggests that there may be multiple factors which contribute to these disparities in these patient populations. Further work to assess the individual factors which contribute to this increased risk, including biologic determinants of disease, socioeconomic status, disease transmission dynamics, and policy practice are needed to assess this highly confounded relationship.

## Conclusion

Our research suggests that there are particularly vulnerable populations that are experiencing a greater proportion of the COVID-19 disease burden, most notably African American and Hispanic individuals, in addition to those living in poverty or in more geographically dense locations. This study has limitations, including its lack of longitudinal data and follow-up, its ecological design, and the lack of specific SDH data. We also note that these findings have limited generalizability outside the United States due to differences in social determinants of health, COVID-19 transmission, care seeking behavior, and healthcare systems. Additionally, differences in care-seeking behavior may have led to variability in testing rate, resulting in a skewed sample. Further studies and interventions are urgently needed to understand and to address the disproportionate burden of COVID-19 in minority communities and when economic disparities are present.

## Data Availability Statement

The raw data supporting the conclusions of this article will be made available by the authors, without undue reservation.

## Ethics Statement

The studies involving human participants were reviewed and approved by Indiana University School of Medicine. Written informed consent from the participants' legal guardian/next of kin was not required to participate in this study in accordance with the national legislation and the institutional requirements.

## Author Contributions

AH conceptualized and designed the study, carried out the initial analysis, drafted the initial manuscript, and reviewed and revised the manuscript. DH conceptualized and designed the study and reviewed and revised the manuscript. AS and MS conceptualized and designed the study, reviewed and revised the manuscript, and critically reviewed the manuscript for important intellectual content. All authors approved the final manuscript as submitted and agreed to be accountable for all aspects of the work.

## Conflict of Interest

The authors declare that the research was conducted in the absence of any commercial or financial relationships that could be construed as a potential conflict of interest.
